# A Case of Abiraterone-Related Hypokalemia Leading to Torsades de Pointes and Cardiac Arrest

**DOI:** 10.7759/cureus.23678

**Published:** 2022-03-31

**Authors:** Dae Hyun Lee, David B Money, Akshay Deshpande, Brian Samuels

**Affiliations:** 1 Cardiology, University of South Florida, Tampa, USA; 2 Internal Medicine, University of South Florida, Tampa, USA; 3 Emergency Medicine, University of South Florida, Tampa, USA

**Keywords:** hypokalemia, cardiotoxicity, cardio-oncology, torsades de pointes, abiraterone

## Abstract

Abiraterone acetate is an androgen-depriving therapy (ADT) that is highly effective for treating castration-resistant prostate cancer (CRPC). By inhibiting CYP17, abiraterone can induce a state of mineralocorticoid excess, which is associated with profound hypokalemia. We present a case of abiraterone-related hypokalemia which led to torsades de pointes (TdP) and ventricular fibrillation (VF). We reviewed the literature and showed the need for close monitoring of the potassium level and electrocardiogram (ECG) to prevent fatal arrhythmias.

## Introduction

Abiraterone is an androgen-depriving therapy (ADT) for treating castration-resistant prostate cancer (CRPC), which works by reducing systemic testosterone [1-4]. It also has a well-known side effect of hypokalemia seen in about 30% because of a reduction in glucocorticoid production [4]. The combination of reduced testosterone and hypokalemia are highly pro-arrhythmogenic, including torsades de pointes (TdP); however, a report of abiraterone-induced TdP is scarce [2, 5-8].

## Case presentation

We report a 61-year-old male with syncope in the oncology clinic. He has a history of metastatic prostate cancer treated with abiraterone and prednisone for one year. Prostate cancer was initially diagnosed in 2012 as limited-stage pT3bN0Mx (Gleason score 3+5). He underwent a prostatectomy with 80% prostate involvement with malignancy, extraprostatic extension into the seminal vesicle, and a positive bladder wall margin. In 2015, he had diffused osteoblastic skeletal metastasis with cord compression. He was on leuprolide acetate (Lupron - leuprolide acetate, AbbVie, Chicago, USA) from 2015 to 2016. In 2016, he developed a castrate-resistant disease and was started on abiraterone when he presented for this hospitalization in 2018.

**Figure 1:**
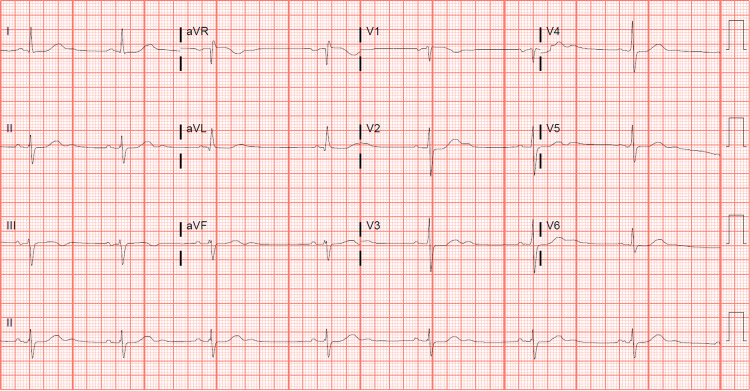
Baseline electrocardiogram.

The patient presented with a sudden episode of syncope while standing up in oncology treatment. He had preceding lightheadedness before syncope with associated post-ictal urinary incontinence. The patient had no chest pain, dyspnea, headache, and neurologic deficits. The rapid response team performed an electrocardiogram (ECG) in the clinic, showing sinus bradycardia with the presence of U-waves and a prolonged QT interval with corrected QT of 540 ms (Bazett's formula) (Figure 1).

**Figure 2:**
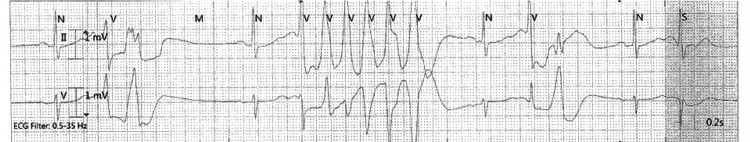
Telemetry of nonsustained VT. VT, ventricular tachycardia

On arrival at the emergency room, he had multiple episodes of non-sustained ventricular tachycardia (VT) (Figure 2).

**Figure 3:**
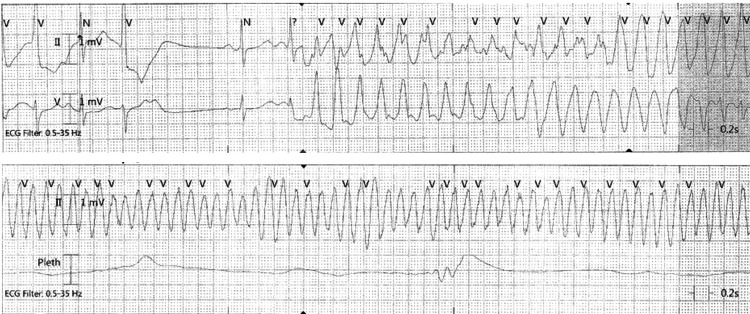
Telemetry of development of TdP and polymorphic VT. TdP, torsades de pointes; VT, ventricular tachycardia

The patient then rapidly progressed to TdP (Figure 3).

Advanced cardiac life support with external defibrillation and chest compression was performed for three minutes. Laboratory tests were done in the emergency room. He was found to be profoundly hypokalemic at 2.4 mEq/L. There were no other medications associated with the development of hypokalemia or TdP. He did not have gastrointestinal symptoms including nausea, vomiting, or diarrhea. Potassium was initially repleted with 60 mEq of oral potassium followed by 40 mEq IV potassium over four hours. He was given an additional 140 mEq IV potassium to maintain potassium levels above 4 mEq/L (final 4.6 mEq/L). He was observed overnight on cardiac telemetry without recurrence of ventricular arrhythmia. Transthoracic echocardiogram did not reveal any structural heart disease. Cardiac stress test with nuclear perfusion imaging was normal without ischemia or infarction as the polymorphic VT could be from ischemic etiology. CT of the head showed no intracranial processes making traumatic brain injury less likely. Upon discharge, his abiraterone and prednisone were restarted, as hypokalemia was a preventable cause of ventricular arrhythmia. He was continued on daily potassium supplementation of 40 mEq. He had no recurrence of ventricular arrhythmias or syncope afterward, with the maintenance of normal potassium levels. 

## Discussion

We describe a case of TdP secondary to hypokalemia from abiraterone use. CRPC is defined as advanced prostate cancer with evidence of disease progression while on appropriate ADT and serum testosterone <50 ng/L. The FDA approved the use of abiraterone acetate (Zytiga - Abiraterone acetate, Janssen Biotech, Beerse, Belgium) with combination prednisone to treat metastatic CRPC in patients who had already received chemotherapy in 2011. In 2012, it was expanded to first-line therapy for metastatic CRPC 2012 with or without prior chemotherapy [9]. Abiraterone plus prednisone, when compared to placebo, showed improved overall survival in the treatment of CRPC and higher incidences of hypertension and hypokalemia (12% and 3%, respectively) [10].

Abiraterone is a progesterone derivative that works by inhibiting CYP17 (17α-hydroxylase/c-17, 20-lyase) which are key enzymes in the biosynthetic pathway of both androgens and glucocorticoids [2, 11]. Abiraterone decreases the androgen supply used by most prostate cancer tumors by inhibiting the conversion of pregnenolone to dehydroepiandrosterone (DHEA) and progesterone to androstenedione. While inhibiting CYP17 effectively reduces circulating serum androgens, it also reduces corticosteroid synthesis by inhibiting the conversion of progesterone to 11-deoxycortisol and cortisol. Inhibiting glucocorticoid production triggers a negative feedback loop, leading to increased adrenocorticotropic hormone (ACTH) production and subsequent mineralocorticoid production [2, 5]. This creates a state of increased mineralocorticoid receptor activity in collecting duct principal cells, which upregulates epithelial Na+ channel (ENaC) expression leading to sodium retention, and potassium efflux through renal outer medullary K+ channel (ROMK) channels. This effect can lead to profound hypokalemia and an arrhythmogenic state.

With hypertension and hypokalemia being well-described side effects of abiraterone in the existing literature, the FDA recommends monthly monitoring of blood pressure and serum potassium while on abiraterone. However, during the past 18 months before the index hospitalization, the patient was not adherent to monthly electrolyte level tests. In the past, potassium blood levels ranged from 2.8 to 3.4 mEq/L. 

A confounding variable, in this case, includes the concurrent treatment with prednisone. Most glucocorticoids have inherent mineralocorticoid properties, and prednisone has a relative mineralocorticoid activity of 0.8 [12]. The utility of prednisone use with abiraterone is to supply the lost glucocorticoid production when CYP17 is inhibited. While a relatively weak effect compared to the mineralocorticoid excess inherently produced by abiraterone, it may be considered given the profound hypokalemia present on admission.

Hypokalemia is defined as a serum K+ less than 3.5 mmol/L [13]. There are numerous causes of hypokalemia, and it is very commonly a side effect of medications. This alone can be an inciting factor of cardiac arrhythmias ranging from premature atrial contractions to VT and ventricular fibrillation (VF). Life-threatening arrhythmias secondary to hypokalemia are more commonly observed with serum potassium levels < 3.0 mol/L [13]. Our patient presented with severe hypokalemia with K of 2.4 mmol/L. Hypokalemia results in hyperpolarization due to an increased potassium gradient across the cell membrane. In most cells, this causes reduced excitability and requires a much stronger stimulus to depolarize. In cardiac myocytes, however, this results in hyperexcitability. Hypokalemia reduces the activity of the Na+/K+-ATPase, which leads to an increase in intracellular Ca+ and thus increases calcium overload resulting in arrhythmias [13].

We believe that the development of TdP was multifactorial through both profound hypokalemia and hypoandrogenism. Hypokalemia causes slowed electrical conduction in cardiac myocytes and prolonged ventricular repolarization, leading to ventricular arrhythmias like TdP [14]. In humans, testosterone shortens the QTc. It increases IKr and IKs channels, thereby increasing the amount of potassium that can leave the cardiac myocyte. Therefore, using ADT will lead to a reduction of testosterone and prolongation of the QTc interval. It has been associated with QTc lengthening anywhere between 10 and 20 ms [2]. Therefore, there is a “double-hit” mechanism of QTc prolongation resulting from hypokalemia and reduced testosterone resulting in arrhythmia.

## Conclusions

In conclusion, we describe a near-fatal case of abiraterone-induced TdP and VF requiring advanced cardiac life support (ACLS) followed by aggressive potassium repletion. Physicians treating patients with abiraterone should be aware of the complications of ADT therapy, namely the anti-androgenic effects and potassium wasting, which can cause near-fatal arrhythmias. There should be close monitoring of potassium levels regularly to prevent life-threatening arrhythmias.
